# Comparative Study of the Dual Layer Magnet Array in a Moving-Coil Tubular Linear PM Motor

**DOI:** 10.3390/s18061854

**Published:** 2018-06-06

**Authors:** Liang Yan, Lu Zhang, Lei Peng, Zongxia Jiao

**Affiliations:** School of Automation Science and Electrical Engineering, Beihang University, Beijing 100191, China; zhanglu@buaa.edu.cn (L.Z.); leipengrun@163.com (L.P.); zxjiao@buaa.edu.cn (Z.J.)

**Keywords:** linear motor, dual layer magnet array, electromagnetic fields, PM machines, comparative study

## Abstract

Conventional single-layer magnet arrays are widely utilized in electromagnetic linear machines. The objective of this paper is to analyze various types of novel dual-layer magnet arrays, either in similar or different patterns, to compare their flux field distribution and increase flux density in the machine. High flux density helps to improve the sensitivity of electromagnetic displacement sensors or actuator thrust. The design concept of magnet arrays are presented. The machine space is divided into several regions according to the magnetic properties. The corresponding magnetic field distribution is formulated based on magnetic vector potential and Laplace’s equations. Numerical computation is conducted to validate the developed magnetic field model. A systematic comparison of magnetic field of various magnet arrays is carried out. It shows that the dual Halbach magnet array can generate relatively high and constant flux density, which may help to produce strong signals. A research prototype and an experimental testbed are developed to validate the analytical model of dual Halbach array. This study provides a general framework for the design and analysis of dual-layer magnet arrays with various magnetization patterns. It can be extended to multiple-layer designs in radial direction.

## 1. Introduction

Permanent magnet linear machines can produce translational motions in high efficiency due to the absence of motion conversion parts such as mechanical gears and screws [1,2,3]. Thus, it is increasingly employed in different fields of applications ranging from transportation, manufacturing to material processing. Moreover, tubular linear machines with permanent magnet excitation are much more attractive compared with flat linear motors due to several distinctive features, such as less end-turn effects, high force density and excellent servo characteristics [4,5,6]. As the development of technologies in industry, the requirement of linear machines’ output performance increases rapidly, especially in aerospace applications where the weight and volume play a crucial role [7,8,9]. High flux density helps to increase force output or signal strength depending on particular tasks [10]. Therefore, magnetic flux density is considered to be an important factor in the design and analysis of linear machines [11,12,13].

Conventionally, three types of magnetization topology have been proposed and analyzed by researchers, i.e., the axial magnetization array, radial magnetization array and Halbach magnetization array [14,15,16]. Hor et al. designed a linear PM machine with radially magnetized magnets [17]. However, radially anisotropic ring magnets are likely to be more expensive and require a custom-designed impulse magnetizing fixture. Conversely, axially magnetized linear PM machines have been widely analyzed because of the advantages of low manufacturing cost and availability of axially anisotropic magnets [18,19,20]. Due to its inherent properties, however, axially magnetization topologies result in high force ripple. By combining axial and radial magnetization topologies, a novel magnetization array named Halbach magnetization array has been proposed by Halbach [21]. This type of magnetization array rapidly attracts the attention of researchers [22,23,24]. Tubular linear PM machines with Halbach magnet patterns have numerous impressive characteristics, such as self-shielding, lower force ripple while its force capability is comparable to those with radial or axial magnetization. Design optimization and comparison of these three topologies in tubular linear PM machines has been conducted by Wang et al. [25]. From this study, it has been shown that for slotless moving-PM machines an axially magnetized topology provides higher force density than that of other two magnetization topologies. However, its high force density is achieved at the expense of larger PM volume. If the magnet volume of axial magnetization reduces to the same size as that of radial and Halbach arrays, the resulting force density is slightly less than that of Halbach topology.

All above studies are based mainly on the analysis of single layer PM array topologies. For further improvement of force density, Yan et al. proposed a dual Halbach array in moving-coil tubular linear permanent magnet machine [26,27,28]. It was shown that the novel array in these studies not only improves significantly the force density but also reduces radial force and vibration disturbance. However, there are several dual layer magnetization arrays by combining any two of the three typical single layer magnetization array aforementioned. These various dual layer magnetization topologies have not been researched and reported before. Therefore, a systematic quantitative comparison has been done in this paper to provide a general framework for the analysis of linear machines with dual layer magnetization. The design concept of typical dual layer magnet patterns are presented. Field distribution of each dual layer array is established analytically based on magnetic vector potential method in cylindrical coordinate system, and the results are verified by finite element analysis. A research prototype and an experimental testbed have been developed, and experimental results are also used to validate the analytical model. The validated analytical field solutions allow the prediction of the flux field of dual layer magnet topologies. A comparative study of flux field for different magnetization topologies is presented. The results can provide an effective tool to choose favorable magnetization pattern for particular designs.

## 2. Topologies of Magnetic Field Distribution

**Figure 1:**
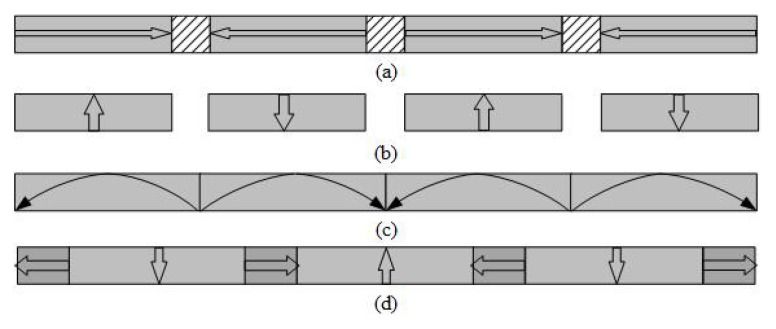
Typical single layer magnet array topologies of tubular linear PM motor: (**a**) axial magnetization; (**b**) radial magnetization; (**c**) Halbach magnetization; and (**d**) quasi-Halbach magnetization.

**Figure 2:**
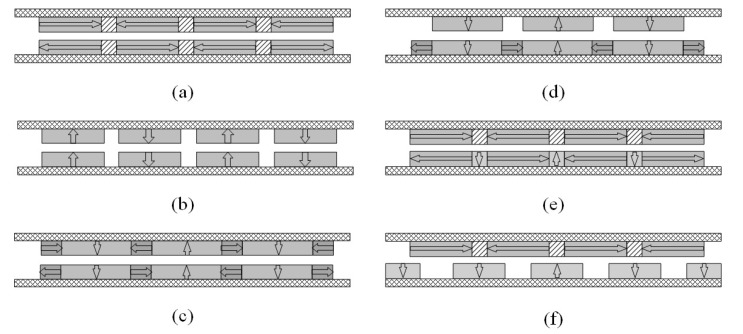
Topologies of double-layer magnetic array: (**a**) dual layer axial magnetization; (**b**) dual layer radial magnetization; (**c**) dual layer quasi-Halbach magnetization; (**d**) dual layer with quasi-Halbach array and radial magnet; (**e**) dual layer with quasi-Halbach magnetization and axial magnet; and (**f**) dual layer with radial magnetization and axial magnetization.

Figure 1 presents three typical types of single layer magnet arrays usually used in tubular linear PM motors, i.e., radial magnetization, axial magnetization and Halbach/quasi-Halbach magnetization. By combining any two of these arrays, variants of double layer magnetic arrays are obtained as shown in Figure 2. Compared with single layer magnet array, its double layer counterparts can enhance magnetic flux density in the air gap more or less. In all these topologies, the armature could be either air- or iron-cored, and either slotless or slotted. Generally, the slotted iron-cored topology has higher force density, but produces undesirable destabilizing tooth ripple cogging force when operating at high speed. On the other hand, the slotless armature structure diminishes tooth ripple cogging effect, and thereby improves the dynamic output performance and servo characteristics at the expense of reduction of force capacity.

Analytical models of magnetic field distribution in the foregoing topologies of dual layer magnet arrays are established with the following assumptions:

(1) The axial length of the motor is infinite along the *z* axis and the motor’s structure is axially symmetric and periodic in the *z* direction. The edge effects associated with the finite length of the motor will be considered by studying a whole model which includes multiple such length-limited motors with enough long distance between any adjacent two motors for convenient Fourier expansion [29].
(2) The permeability of the iron is infinite, which means saturation is ignored.
(3) The armature is slotless. However, slotting effects, if present, can be taken into account by introducing a Carter coefficient [30].

**Figure 3:**
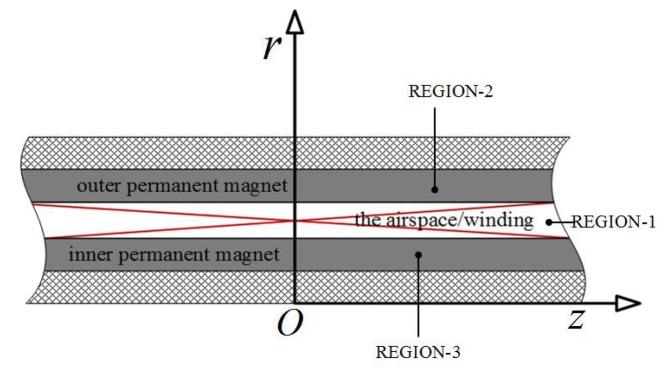
Three solving regions of the magnetic field analysis.

According to the magnetic permeability of materials, the solving region of the flux field is divided into three regions as shown in Figure 3.

The flux density in these three regions is characterized by

(1) $$\begin{array}{cc} B=\mu_{0}H, & \mathrm{Airspace}\\ B=\mu_{0}\mu_{r}H+\mu_{0}M, & \mathrm{Inner}/\mathrm{Outer}-\mathrm{PM} \end{array}$$

where $\mu_{r}$ is the relative recoil permeability of magnets and **M** is the magnetization intensity of magnet. For a permanent magnet that has a linear demagnetization characteristic, $\mu_{r}$ is constant and the remanent magnetization **M** is related to the remanence **$B_{rem}$** by

(2) $$\begin{array}{c} M=B_{rem}/\mu_{0}. \end{array}\;$$

According to the inherent solenoid characteristic of magnetic field, the flux density **B** satisfies

(3) $$\begin{array}{c} \nabla \cdot B=0. \end{array}$$

For convenience of derivation, the magnetic vector potential **A** is introduced in terms of the Coulomb gauge as

(4) $$\begin{array}{c} B=\nabla \times A. \end{array}$$

Thus, the governing equations of magnetic field based on Maxwell differential equation are

(5) $$\begin{array}{cc} \nabla^{2}A_{I}=0, & \mathrm{Airspace}(\mathrm{Region}1)\\ \nabla^{2}A_{\mathrm{II}}=-\mu_{0}\nabla \times M, & \mathrm{Outer}-\mathrm{PM}(\mathrm{Region}2)\\ \nabla^{2}A_{\mathrm{III}}=-\mu_{0}\nabla \times M, & \mathrm{Inner}-\mathrm{PM}(\mathrm{Region}3) \end{array}$$

where the first equation is a Laplace equation and the last two equations are Poisson equations. In cylindrical coordinate system, **A** can be decomposed as $A=A_{r}e_{r}+A_{\theta }e_{\theta }+A_{z}e_{z}$ and **M** is given by

(6) $$\begin{array}{c} M=M_{r}e_{r}+M_{z}e_{z}.\; \end{array}$$

Since the field is axially symmetric, **A** only has the component of $A_{\theta }$ that is independent of θ. The following Equation (7) includes governing equations of Regions 1, 2 and 3, respectively.

(7) $$\begin{array}{c} \frac{\partial }{\partial r}\left(\frac{1}{r}\frac{\partial }{\partial r}\left(rA_{I\theta }\right)\right)+\frac{\partial^{2}A_{I\theta }}{\partial z^{2}}=0,\\ \frac{\partial }{\partial r}\left(\frac{1}{r}\frac{\partial }{\partial r}\left(rA_{\mathrm{II}\theta }\right)\right)+\frac{\partial^{2}A_{\mathrm{II}\theta }}{\partial z^{2}}=-\mu_{0}\nabla \times M,\\ \frac{\partial }{\partial r}\left(\frac{1}{r}\frac{\partial }{\partial r}\left(rA_{\mathrm{III}\theta }\right)\right)+\frac{\partial^{2}A_{\mathrm{III}\theta }}{\partial z^{2}}=-\mu_{0}\nabla \times M. \end{array}$$

The flux density components are deduced from $A_{\theta }$ by

(8) $$\begin{array}{c} B_{z}=\frac{1}{r}\frac{\partial }{\partial r}\left(rA_{\theta }\right),\\ B_{r}=-\frac{\partial A_{\theta }}{\partial z}. \end{array}$$

The solution form to Equation (7) depends on the specific magnet array topology, each of which will be considered separately as follows.

### 2.1. Dual Layer Halbach Magnetization Topology

**Figure 4:**
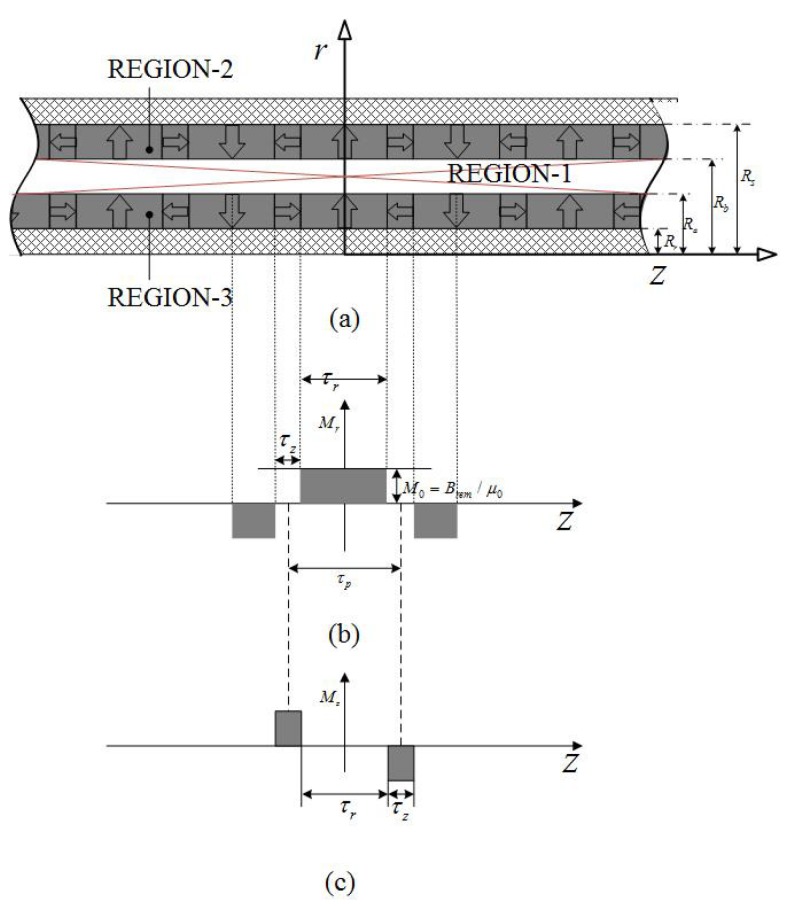
Field regions of dual layer quasi-Halbach magnetization array topology: (**a**) solving regions, (**b**,**c**) magnetization distributions.

Figure 4 presents the simplified model of the double Halbach magnet array topology in which the magnetization, **M**, is given by

(9) $$\begin{array}{cc} M=M_{r}e_{r}+M_{z}e_{z}, & \mathrm{Inner}\mathrm{PM}\\ M=M_{r}e_{r}-M_{z}e_{z}. & \mathrm{Outer}\mathrm{PM} \end{array}$$

By using Fourier expansion, $M_{r}$ and $M_{z}$ can be expressed as

(10) $$\begin{array}{c} M_{r}=\sum_{n=1}^{\infty }\frac{4B_{rem}}{\left(2n\;-\;1\right)\pi \mu_{0}}\sin\left(\frac{\left(2n\;-\;1\right)\pi \tau_{r}}{2\tau_{p}}\right)\cos\left(m_{n}z\right),\\ M_{z}=-\sum_{n=1}^{\infty }\frac{4B_{rem}}{\left(2n\;-\;1\right)\pi \mu_{0}}\cos\left[\frac{\left(2n\;-\;1\right)\pi \tau_{r}}{2\tau_{p}}\right]\sin\left(m_{n}z\right), \end{array}$$

where $\tau_{r}$ is pole-length, $\tau_{p}$ is pole-pitch and $m_{n}=\left(2n\;-\;1\right)\pi /\tau_{p}$. Combining Equations (7) and (10) yields

(11) $$\begin{array}{c} \frac{\partial }{\partial r}\left(\frac{1}{r}\frac{\partial }{\partial r}\left(rA_{I\theta }\right)\right)+\frac{\partial^{2}A_{I\theta }}{\partial z^{2}}=0,\\ \frac{\partial }{\partial r}\left(\frac{1}{r}\frac{\partial }{\partial r}\left(rA_{\mathrm{II}\theta }\right)\right)+\frac{\partial^{2}A_{\mathrm{II}\theta }}{\partial z^{2}}=\sum_{n=1,2,\ldots }^{\infty }P_{n}\sin\left(m_{n}z\right),\\ \frac{\partial }{\partial r}\left(\frac{1}{r}\frac{\partial }{\partial r}\left(rA_{\mathrm{III}\theta }\right)\right)+\frac{\partial^{2}A_{\mathrm{III}\theta }}{\partial z^{2}}=\sum_{n=1,2,\ldots }^{\infty }P_{n}\sin\left(m_{n}z\right), \end{array}\;$$

where $P_{n}=\frac{4B_{rem}}{\tau_{p}}\sin\left(\frac{\left(2n\;-\;1\right)\pi \tau_{r}}{2\tau_{p}}\right)$. The boundary conditions of the solution to Equation (11) are

(12) $$\begin{array}{c} H_{\mathrm{III}z}{|}_{r=R_{r}}=0;H_{\mathrm{II}z}{|}_{r=R_{s}}=0,\\ H_{\mathrm{III}z}{|}_{r=R_{a}}=H_{Iz}{|}_{r=R_{a}};B_{\mathrm{III}r}{|}_{r=R_{a}}=B_{Ir}{|}_{r=R_{a}},\\ H_{\mathrm{II}z}{|}_{r=R_{b}}=H_{Iz}{|}_{r=R_{b}};B_{\mathrm{II}r}{|}_{r=R_{b}}=B_{Ir}{|}_{r=R_{b}}. \end{array}$$

As a result, the general solutions to Equation (11) are flux density in the airspace/winding (Region-1)

(13) $$\begin{array}{c} B_{Ir}=\sum_{n=1}^{\infty }-m_{n}\left[a_{In}I_{1}\left(m_{n}r\right)+b_{In}K_{1}\left(m_{n}r\right)\right]\cos\left(m_{n}z\right),\\ B_{Iz}=\sum_{n=1}^{\infty }m_{n}\left[a_{In}I_{0}\left(m_{n}r\right)-b_{In}K_{0}\left(m_{n}r\right)\right]\sin\left(m_{n}z\right). \end{array}\;$$

The flux density in the outer PM region (Region-2) is

(14) $$\begin{array}{c} B_{\mathrm{II}r}=-\sum_{n=1}^{\infty }m_{n}\left\{\begin{array}{c} \left[a_{\mathrm{II}n}I_{1}\left(m_{n}r\right)+b_{\mathrm{II}n}K_{1}\left(m_{n}r\right)\right]\cos\left(m_{n}z\right)\\ +\frac{\pi L_{1}\left(m_{n}r\right)}{2{m_{n}}^{2}}P_{n}\cos\left(m_{n}z\right) \end{array}\right\},\\ B_{\mathrm{II}z}=\sum_{n=1}^{\infty }m_{n}\left\{\begin{array}{c} \left[a_{\mathrm{II}n}I_{0}\left(m_{n}r\right)-b_{\mathrm{II}n}K_{0}\left(m_{n}r\right)\right]\sin\left(m_{n}z\right)\\ +\frac{\pi L_{0}\left(m_{n}r\right)}{2{m_{n}}^{2}}P_{n}\sin\left(m_{n}z\right) \end{array}\right\}. \end{array}$$

Flux density in the inner PM region (Region-3) is

(15) $$\begin{array}{c} B_{\mathrm{III}r}=-\sum_{n=1}^{\infty }m_{n}\left\{\begin{array}{c} \left[a_{\mathrm{III}n}I_{1}\left(m_{n}r\right)+b_{\mathrm{III}n}K_{1}\left(m_{n}r\right)\right]\cos\left(m_{n}z\right)\\ +\frac{\pi L_{1}\left(m_{n}r\right)}{2{m_{n}}^{2}}P_{n}\cos\left(m_{n}z\right) \end{array}\right\},\\ B_{\mathrm{III}z}=\sum_{n=1}^{\infty }m_{n}\left\{\begin{array}{c} \left[a_{\mathrm{III}n}I_{0}\left(m_{n}r\right)-b_{\mathrm{III}n}K_{0}\left(m_{n}r\right)\right]\sin\left(m_{n}z\right)\\ +\frac{\pi L_{0}\left(m_{n}r\right)}{2{m_{n}}^{2}}P_{n}\sin\left(m_{n}z\right) \end{array}\right\}. \end{array}\;$$

### 2.2. Dual Layer Radial Magnetization Topology

**Figure 5:**
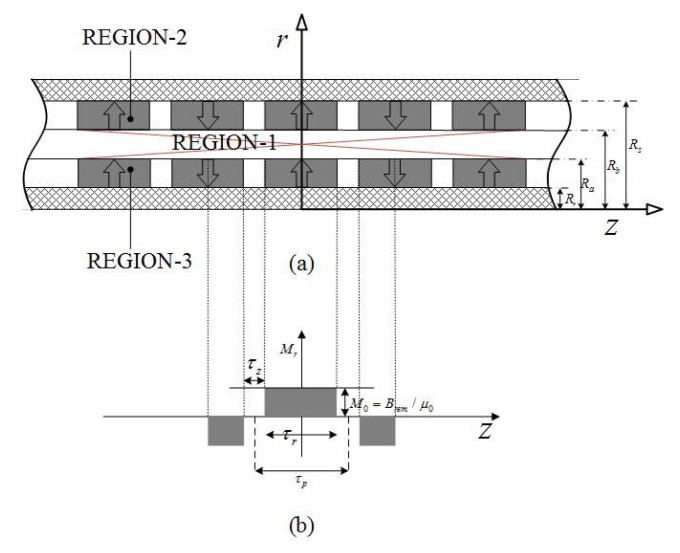
Filed regions of dual layer radially magnetized array topology: (**a**) field regions and (**b**) magnetization distributions.

The simplified model of the double layer radial magnetization topology is shown in Figure 5. $M=M_{r}e_{r}$ for both inner and outer PM regions. Thus, the three components of ∇×M can be expressed as

(16) $$\begin{array}{c} \nabla \times M=\left\{\begin{array}{c} \left(\frac{1}{r}\frac{\partial M_{z}}{\partial \theta }-\frac{\partial M_{\theta }}{\partial z}\right)=0\\ \frac{\partial M_{r}}{\partial z}-\frac{\partial M_{z}}{\partial r}=\frac{\partial M_{r}}{\partial z}\\ \frac{1}{r}\left(\frac{\partial \left(rM_{\theta }\right)}{\partial r}-\frac{\partial M_{r}}{\partial \theta }\right)=0 \end{array}\right. \end{array}.$$

The governing equation is derived as same as that of dual Halbach magnet array in Equation (11). Furthermore, the permeability of air and magnet is very close to each other. Thus, the airspace between two adjacent radial magnets can be considered as an equivalent PM whose magnetization **M** is zero. As a result, the boundary conditions are the same as Equation (12). In other words, the governing field equations and the boundary conditions are completely the same as the ones of dual Halbach magnetic array. The flux density distributions are thus given by Equations (13)–(15) similarly.

### 2.3. Dual Layer Axial Magnetization Topology

**Figure 6:**
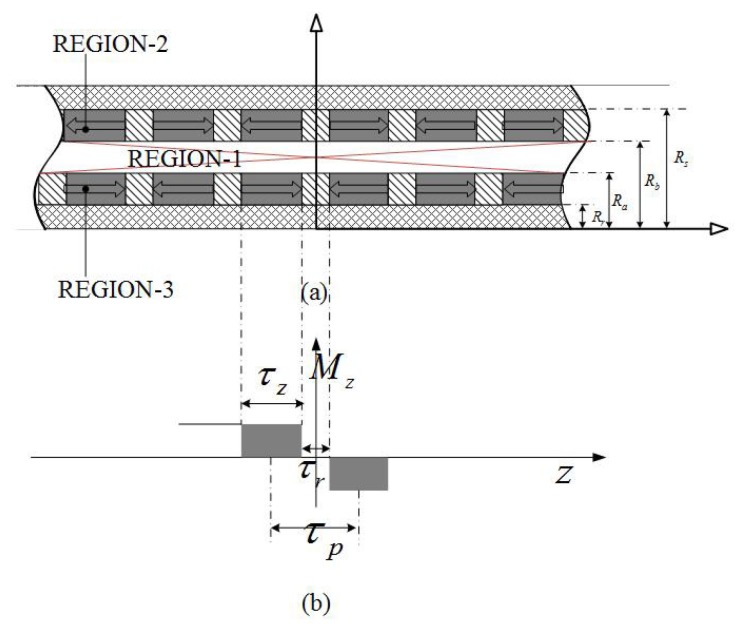
Filed regions of dual layer axially magnetized array topology: (**a**) field regions and (**b**) magnetization distributions.

Figure 6a shows the simplified model of dual axial magnet array topology where the magnets are separated with infinite permeable iron pole pieces. The magnetization vector is described by

(17) $$\begin{array}{cc} M=M_{z}e_{z}, & \mathrm{Inner}\mathrm{PM}\\ M=-M_{z}e_{z}, & \mathrm{Outer}\mathrm{PM}. \end{array}$$

Due to the absence of $M_{r}$, the governing field equations are simplified and given by

(18) $$\begin{array}{c} \frac{\partial }{\partial r}\left(\frac{1}{r}\frac{\partial }{\partial r}\left(rA_{i\theta }\right)\right)+\frac{\partial^{2}A_{i\theta }}{\partial z^{2}}=0, \end{array}$$

where *i* = I, II and III. The boundary conditions are

(19) $$\begin{array}{c} H_{\mathrm{III}z}{|}_{r=R_{r}}=0;H_{\mathrm{II}z}{|}_{r=R_{s}}=0;\\ H_{\mathrm{III}r}{|}_{z=\pm \tau_{p}/2}=0;H_{\mathrm{II}r}{|}_{z=\pm \tau_{p}/2}=0;\\ H_{Iz}{{|}_{-\tau_{p}/2<z<\tau_{p}/2}^{r=R_{a}}=0;H_{Iz}|}_{-\tau_{p}/2<z<\tau_{p}/2}^{r=R_{b}}=0;\\ H_{\mathrm{III}z}{{|}_{\tau_{p}/2<z<\tau_{p}/2+\tau_{m}}^{r=R_{a}}=H_{Iz}|}_{\tau_{p}/2<z<\tau_{p}/2+\tau_{m}}^{r=R_{a}};\\ B_{\mathrm{III}r}{{|}_{\tau_{p}/2<z<\tau_{p}/2+\tau_{m}}^{r=R_{a}}=B_{Ir}|}_{\tau_{p}/2<z<\tau_{p}/2+\tau_{m}}^{r=R_{a}};\\ H_{\mathrm{II}z}{{|}_{\tau_{p}/2<z<\tau_{p}/2+\tau_{m}}^{r=R_{b}}=H_{Iz}|}_{\tau_{p}/2<z<\tau_{p}/2+\tau_{m}}^{r=R_{b}};\\ B_{\mathrm{II}r}{{|}_{\tau_{p}/2<z<\tau_{p}/2+\tau_{m}}^{r=R_{b}}=B_{Ir}|}_{\tau_{p}/2<z<\tau_{p}/2+\tau_{m}}^{r=R_{b}}; \end{array}$$

The solutions in all three regions that satisfy the boundary conditions in Equation (19) are

(20) $$\begin{array}{c} B_{ir}=\sum_{n=1}^{\infty }-m_{n}\left[a_{in}I_{1}\left(m_{n}r\right)+b_{in}K_{1}\left(m_{n}r\right)\right]\cos\left(m_{n}z\right)\\ B_{iz}=\sum_{n=1}^{\infty }m_{n}\left[a_{in}I_{0}\left(m_{n}r\right)-b_{in}K_{0}\left(m_{n}r\right)\right]\sin\left(m_{n}z\right), \end{array}$$

where i=I,II,III. The determination of $a_{In}$, $b_{In}$, $a_{\mathrm{II}n}$, $b_{In}$, $a_{\mathrm{III}n}$, $b_{\mathrm{III}n}$ is more difficult due to the complexity of the boundary conditions [31].

### 2.4. Axial-Halbach Magnetization Topology

**Figure 7:**
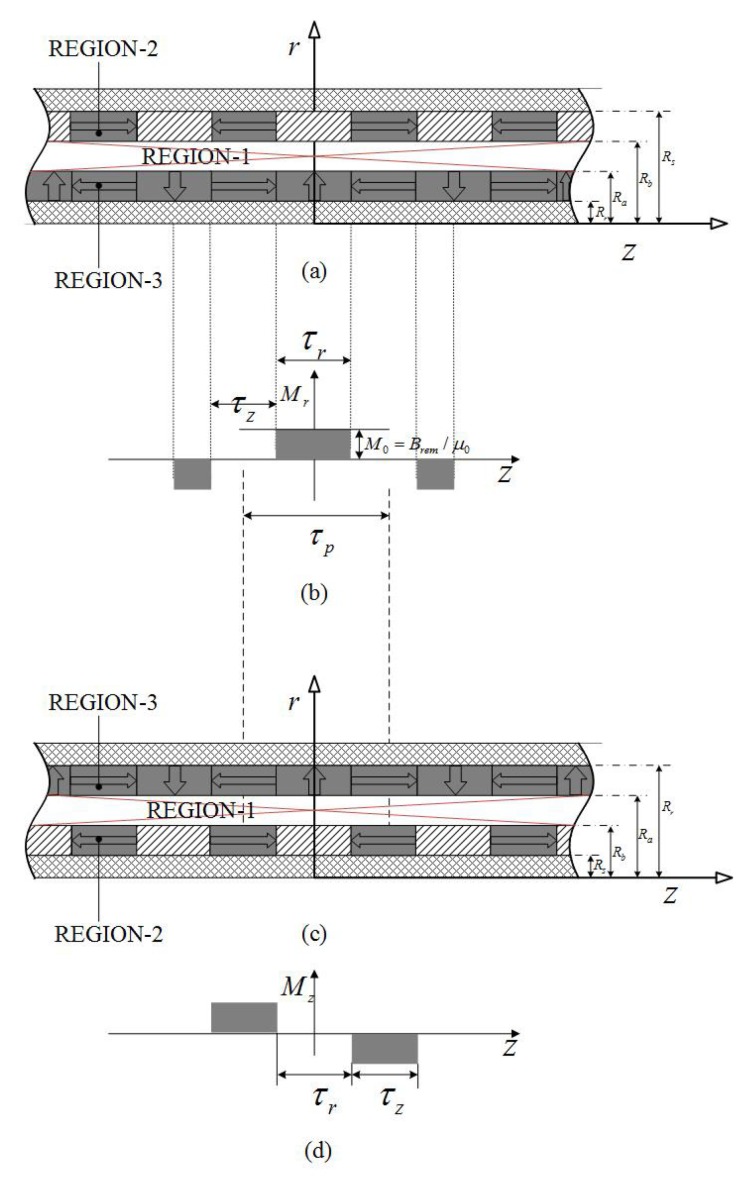
Field regions of dual layer axial-Halbach magnetization topologies: (**a**,**c**) solving regions of two topologies; (**b**,**d**) magnetization distributions.

This topology can be divided into two types as shown in Figure 7 according to the position of magnetic arrays. One type includes axial magnetization as the internal layer shown in Figure 7c, while the other a quasi-Halbach as internal layer shown in Figure 7a. A general framework is set up to analyze these two topologies conveniently. The magnetization in Figure 7b,d, is therefore given by

(21) $$\begin{array}{c} M=\left\{\begin{array}{cc} M_{r}e_{r}+M_{z}e_{z} & \mathrm{Inner}\mathrm{PM}\\ -M_{z}e_{z} & \mathrm{Outer}\mathrm{PM} \end{array}\right. \end{array},$$

or

(22) $$\begin{array}{c} M=\left\{\begin{array}{cc} M_{z}e_{z} & \mathrm{Inner}\mathrm{PM}\\ M_{r}e_{r}-M_{z}e_{z} & \mathrm{Outer}\mathrm{PM} \end{array}\right. \end{array}.$$

Hence, the governing equations of fields in Region 1, 2 and 3 can be obtained as

(23) $$\begin{array}{c} \frac{\partial }{\partial r}\left(\frac{1}{r}\frac{\partial }{\partial r}\left(rA_{I\theta }\right)\right)+\frac{\partial^{2}A_{I\theta }}{\partial z^{2}}=0,\\ \frac{\partial }{\partial r}\left(\frac{1}{r}\frac{\partial }{\partial r}\left(rA_{\mathrm{II}\theta }\right)\right)+\frac{\partial^{2}A_{\mathrm{II}\theta }}{\partial z^{2}}=0,\\ \frac{\partial }{\partial r}\left(\frac{1}{r}\frac{\partial }{\partial r}\left(rA_{\mathrm{III}\theta }\right)\right)+\frac{\partial^{2}A_{\mathrm{III}\theta }}{\partial z^{2}}=\sum_{n=1,2\ldots }^{\infty }P_{n}\sin\left(m_{n}z\right). \end{array}$$

The boundary conditions are

(24) $$\begin{array}{c} \begin{array}{c} H_{\mathrm{III}z}{|}_{r=R_{r}}=0;H_{\mathrm{II}z}{|}_{r=R_{s}}=0;\\ H_{\mathrm{III}z}{|}_{r=R_{a}}=H_{Iz}{|}_{r=R_{a}};\\ H_{\mathrm{II}r}{|}_{z=\pm \tau_{p}/2}=0;H_{Iz}{|}_{-\tau_{p}/2<z<\tau_{p}/2}^{r=R_{b}}=0;\\ H_{\mathrm{II}z}{{|}_{\tau_{p}/2<z<\tau_{p}/2+\tau_{m}}^{r=R_{b}}=H_{Iz}|}_{\tau_{p}/2<z<\tau_{p}/2+\tau_{m}}^{r=R_{b}};\\ B_{\mathrm{II}r}{{|}_{\tau_{p}/2<z<\tau_{p}/2+\tau_{m}}^{r=R_{b}}=B_{Ir}|}_{\tau_{p}/2<z<\tau_{p}/2+\tau_{m}}^{r=R_{b}} \end{array} \end{array}$$

Substituting Equation (23) into Equation (24) yields

(25) $$\begin{array}{c} B_{Ir}=\sum_{n=1}^{\infty }-m_{n}\left[a_{In}I_{1}\left(m_{n}r\right)+b_{In}K_{1}\left(m_{n}r\right)\right]\cos\left(m_{n}z\right),\\ B_{Iz}=\sum_{n=1}^{\infty }m_{n}\left[a_{In}I_{0}\left(m_{n}r\right)-b_{In}K_{0}\left(m_{n}r\right)\right]\sin\left(m_{n}z\right),\\ B_{\mathrm{II}r}=-\sum_{n=1}^{\infty }m_{n}\left\{\left[a_{\mathrm{II}n}I_{1}\left(m_{n}r\right)+b_{\mathrm{II}n}K_{1}\left(m_{n}r\right)\right]\cos\left(m_{n}z\right)\right\},\\ B_{\mathrm{II}z}=\sum_{n=1}^{\infty }m_{n}\left\{\left[a_{\mathrm{II}n}I_{0}\left(m_{n}r\right)-b_{\mathrm{II}n}K_{0}\left(m_{n}r\right)\right]\sin\left(m_{n}z\right)\right\},\\ B_{\mathrm{III}r}=-\sum_{n=1}^{\infty }m_{n}\left\{\begin{array}{c} \left[a_{\mathrm{III}n}I_{1}\left(m_{n}r\right)+b_{\mathrm{III}n}K_{1}\left(m_{n}r\right)\right]\cos\left(m_{n}z\right)\\ +\frac{\pi L_{1}\left(m_{n}r\right)}{2{m_{n}}^{2}}P_{n}\cos\left(m_{n}z\right) \end{array}\right\},\\ B_{\mathrm{III}z}=\sum_{n=1}^{\infty }m_{n}\left\{\begin{array}{c} \left[a_{\mathrm{III}n}I_{0}\left(m_{n}r\right)-b_{\mathrm{III}n}K_{0}\left(m_{n}r\right)\right]\sin\left(m_{n}z\right)\\ +\frac{\pi L_{0}\left(m_{n}r\right)}{2{m_{n}}^{2}}P_{n}\sin\left(m_{n}z\right). \end{array}\right\} \end{array}$$

### 2.5. Radial-Halbach Magnetization

**Figure 8:**
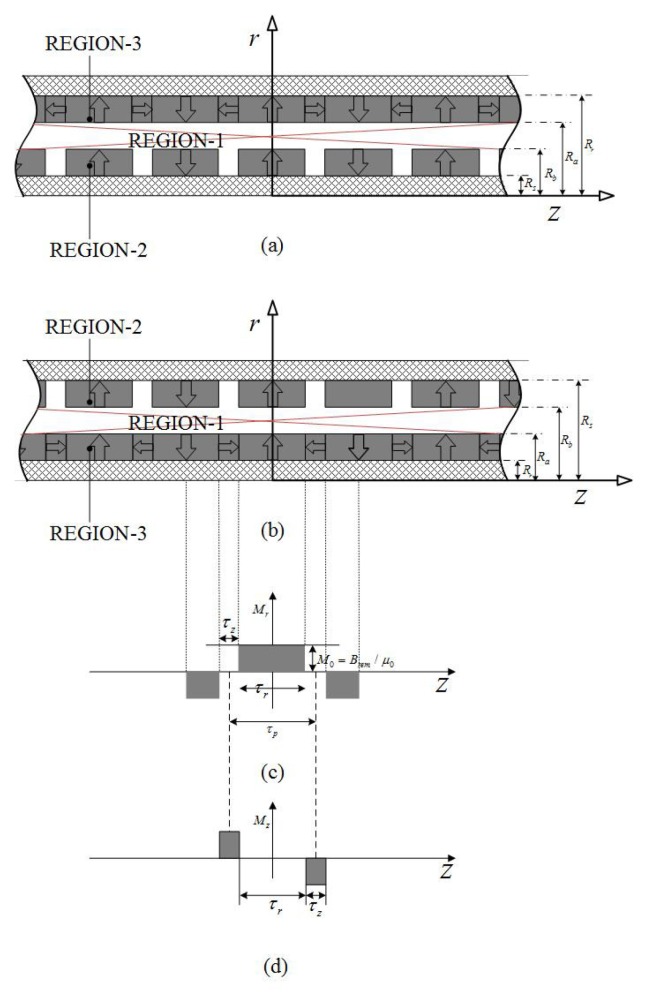
Field regions of dual layer radial-Halbach magnetization topologies: (**a**,**b**) solving regions of two topologies, (**c**,**d**) magnetization distributions.

Radial-Halbach dual layer magnet array can be divided into two types as shown in Figure 8. Thus, the magnetization vector **M** is given by

(26) $$\begin{array}{c} M=\left\{\begin{array}{cc} M_{r}e_{r}+M_{z}e_{z} & \mathrm{Inner}\mathrm{PM}\\ M_{r}e_{r} & \mathrm{Outer}\mathrm{PM} \end{array}\right. \end{array}$$

or

(27) $$\begin{array}{c} M=\left\{\begin{array}{cc} M_{r}e_{r} & \mathrm{Inner}\mathrm{PM}\\ M_{r}e_{r}-M_{z}e_{z} & \mathrm{Outer}\mathrm{PM} \end{array}\right. \end{array}$$

The governing equations and the boundary conditions are derived as the same as Equations (11) and (12), respectively. Thus, the formulation of flux density is the same as Equations (13)–(15) as well.

### 2.6. Axial-Radial Magnetization Topology

**Figure 9:**
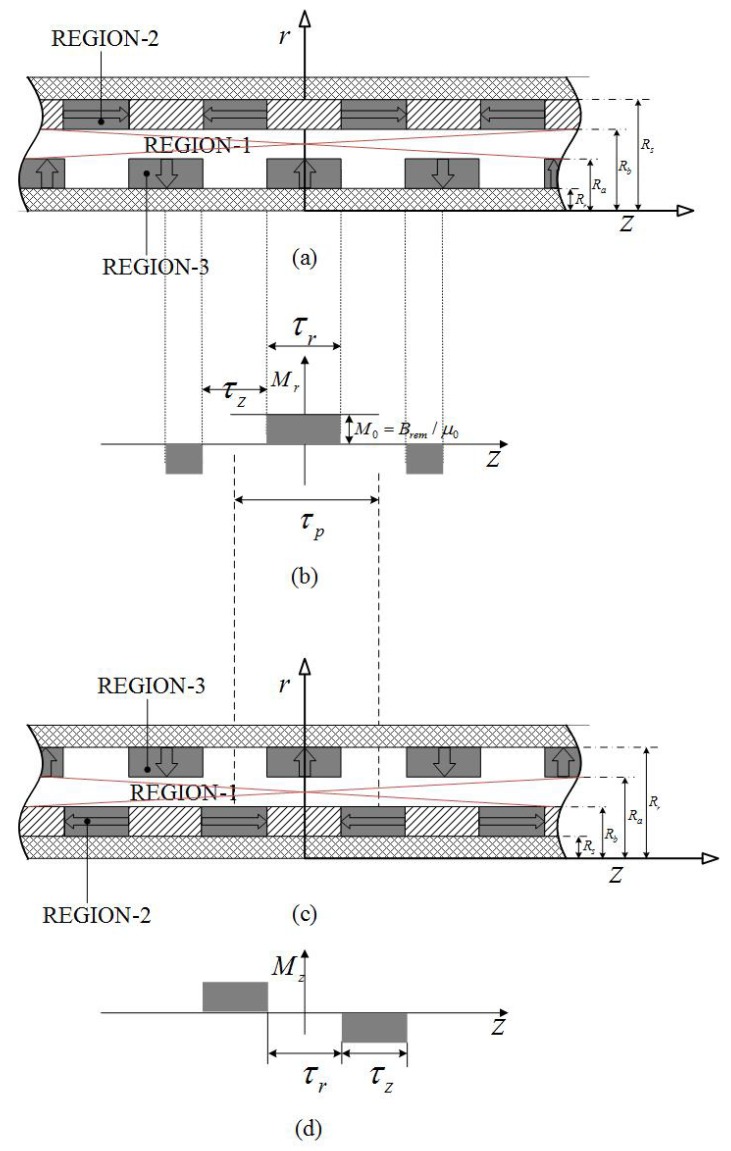
Field regions of dual layer axial-radial magnetization topologies: (**a**,**c**) solving regions of two topologies; (**b**,**d**) magnetization distributions.

Figure 9 shows the simplified models of the two topologies of the axial-radial magnetization. The structure parameters have been adjusted according to the place of magnets in order to obtain a uniform model. The magnetization pattern of the topology is given by

(28) $$\begin{array}{c} M=\left\{\begin{array}{cc} M_{z}e_{z} & \mathrm{Inner}\mathrm{PM}\\ M_{r}e_{r} & \mathrm{Outer}\mathrm{PM} \end{array}\right. \end{array}$$

or

(29) $$\begin{array}{c} M=\left\{\begin{array}{cc} M_{r}e_{r} & \mathrm{Inner}\mathrm{PM}\\ -M_{z}e_{z} & \mathrm{Outer}\mathrm{PM} \end{array}\right. \end{array}$$

According to the approximation mentioned in Section 2.1, the governing equations of axial-radial magnetization can be described with Equation (23). Similarly, the boundary conditions are the same as those of axial-Halbach magnetization topology, i.e., Equation (24). While, the component $M_{z}$ of Halbach magnetization makes $a_{In}$, $b_{In}$, $a_{\mathrm{II}n}$, $b_{In}$, $a_{\mathrm{III}n}$ and $b_{\mathrm{III}n}$ completely different from those of Equation (23). Thereby, a different solution of flux density is obtained.

**Figure 10:**
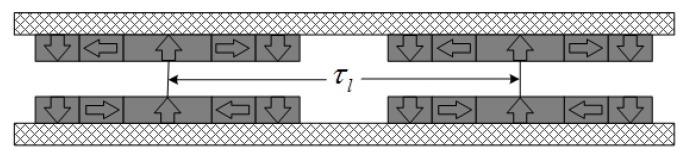
Topology of fresh model which is established for analysis of edgy effects of dual layer quasi-Halbach magnetization array.

To take edge effects into account, one magnet pole-pair and two fringe magnets of the whole magnetic array model are considered as one unit. The novel unit is expanded along the *z* axis with enough distance between neighboring unit structures, e.g., a new model of dual layer Halbach magnetization topology shown in Figure 10. The Fourier expansion of the magnetization vector becomes different from previous one, due to the change of period. Therefore, the two components can be expressed as

(30) $$\begin{array}{c} M_{r}=\frac{2B_{rem}}{n\pi \mu_{0}}\sum_{n=1}^{\infty }\left\{\begin{array}{c} sin\left(m_{n}\frac{\tau_{r}}{2}\right)-sin\left(m_{n}\tau_{p}\right)\\ +sin\left[m_{n}\left(\tau_{p}-\frac{\tau_{r}}{2}\right)\right] \end{array}\right\}\cos\left(m_{n}z\right),\\ M_{z}=\sum_{n=1}^{\infty }\frac{2B_{rem}}{n\pi \mu_{0}}\left[\cos\left(m_{n}\left(\tau_{p}-\frac{\tau_{r}}{2}\right)\right)-\cos\left(m_{n}\frac{\tau_{r}}{2}\right)\right]\sin\left(m_{n}z\right), \end{array}$$

where $m_{n}=n\pi /\tau_{l}$. In the same way, ∇×M is given by

(31) $$\begin{array}{c} \nabla \times M=\left\{\begin{array}{c} \left(\frac{1}{r}\frac{\partial M_{z}}{\partial \theta }-\frac{\partial M_{\theta }}{\partial z}\right)=0\\ \frac{\partial M_{r}}{\partial z}-\frac{\partial M_{z}}{\partial r}=\frac{\partial M_{r}}{\partial z}=\sum_{n-1}^{\infty }P_{n}\sin\left(m_{n}z\right)\\ \frac{1}{r}\left(\frac{\partial \left(rM_{\theta }\right)}{\partial r}-\frac{\partial M_{r}}{\partial \theta }\right)=0 \end{array}\right. \end{array}$$

where

(32) $$\begin{array}{c} P_{n}=\frac{2B_{rem}}{\tau_{l}}\left\{\begin{array}{c} \sin\left(m_{n}\frac{\tau_{r}}{2}\right)-\sin\left(m_{n}\tau_{p}\right)\\ +\sin\left[m_{n}\left(\tau_{p}-\frac{\tau_{r}}{2}\right)\right] \end{array}\right\}. \end{array}$$

Therefore, the boundary conditions and the solutions of flux density by considering the edge effects can be obtained in the same way aforementioned in Section 2.4.

## 3. Numerical Simulation and Experiments

In this section, a comparative study on the radial flux density of six dual layer magnetization arrays is conducted with numerical simulations. According to the results of comparison, dual layer Halbach magnetization array is considered to be a desired option because of the high radial flux density. Therefore, a research prototype of the linear motor with dual Halbach array and an experimental apparatus have been developed. Experimental works on magnetic flux field is conducted to validate the derived analytical model.

### 3.1. Magnetic Field Variation of the Topologies

**Figure 11:**
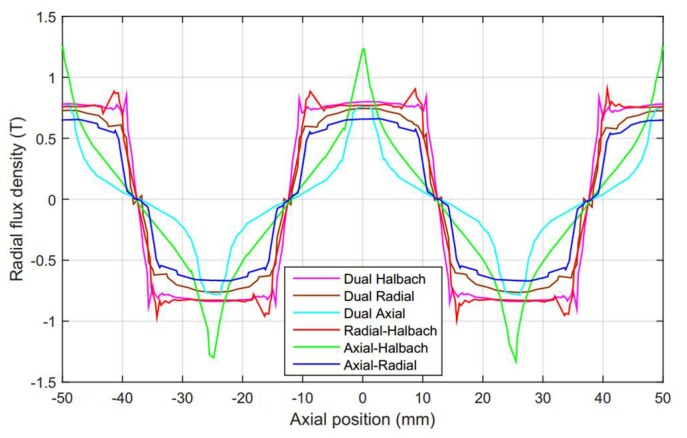
Magnetic field comparative results of radial flux density for these six topologies.

Numerical computation is conducted on the magnetic field distribution of the six magnet topologies. The radial flux component is used to generate axial force, whereas the axial component does not contribute to it. The radial flux field variation of six topologies versus axial distance *z* at the center of air gap, i.e., $r=(R_{a}+R_{b})/2$, is shown in Figure 11. It is found that the average radial flux density of dual layer Halbach magnetization is higher than that of others, i.e., the radial flux curve contains the largest area than other curves. In another way, the peak value of dual Halbach magnetization is higher than dual radial, axial-radial and dual axial topologies. In addition, compared with radial-Halbach and axial-Halbach topologies, the radial flux density of dual Halbach array is more constant, which will produce lowest fluctuation of thrust within stroke. Therefore, the dual Halbach topology is proved to be a desired option for the design of tubular linear machines. It is used for the development of our research prototype.

### 3.2. Prototype and Experimental Apparatus

**Figure 12:**
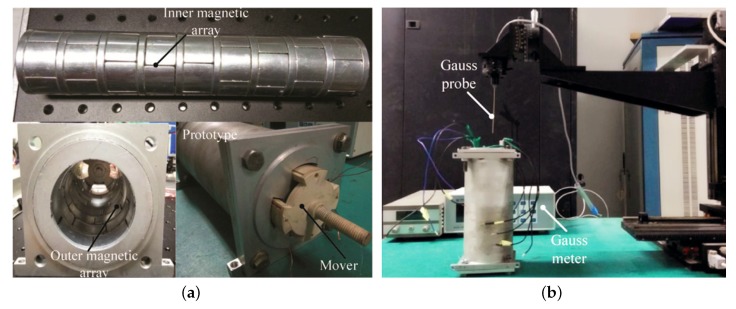
Linear machines with dual Halbach array. (**a**) Research prototype; (**b**) Experimental testbed.

A tubular linear motor with dual Halbach array is developed for experimental investigation on the magnetic field as shown in Figure 12a. Two sets of PM arrays, i.e., the inner and outer magnet arrays, are mounted on the stator. One mover is installed in between these two PM arrays, and winded with several coils. The interaction between the current input in the coils and the the magnetic flux density of PM arrays generates force along the machine axis. The major design parameters of the research prototype are listed in Table 1.

In addition, an experimental apparatus is developed for measurement of magnetic flux density as shown in Figure 12b. A gauss probe is mounted on the end-effort of a three-axis translational platform. The gauss probe can pinpoint into the linear motor and measure the magnetic flux density subsequently. Then, the measured data can be translated to personal computer. After completing the measurement at one particular position, the gauss meter will send signal to the personal computer, and the probe will be moved to the next point automatically by the translational stage.

### 3.3. Validation of Analytical Magnetic Field Model

**Figure 13:**
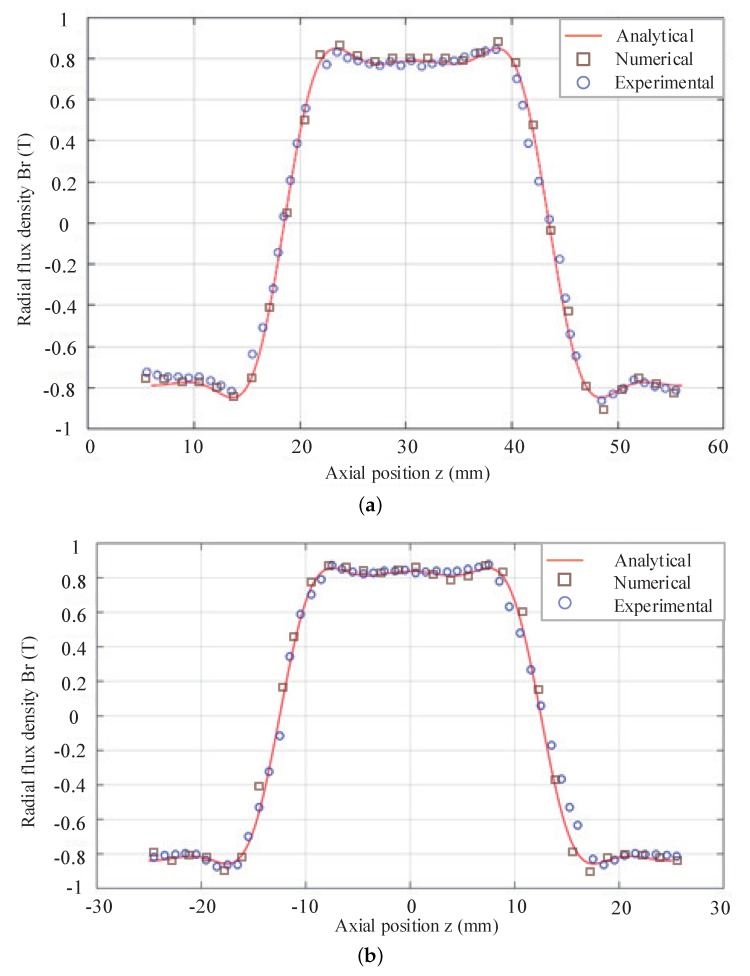
Magnetic field variation versus *z*. (**a**) *r* = 23.5 mm; (**b**) *r* = 31.5 mm.

Experimental investigation is conducted on the developed research prototype with the testbed to validate the analytical magnetic field model. Furthermore, the numerical computation is conducted once again for the validation. The analytical model, numerical result and experiments are all presented in Figure 13. It shows the magnetic field variation in the internal air gap (*r* = 23.5 mm) and external air gap (*r* = 31.5 mm), respectively. It is found that the analytical result agrees with the numerical simulation and experimental measurements well, and thus it could be employed for the design and control implementation of linear machine subsequently. Because of manufacturing and assembly errors, there is some difference among analytical results, numerical results and experimental results. The accuracy of the FEM, analytical method and experimental method is shown in Table 2. Eight data about magnetic field variation are randomly selected in the external air (*r* = 23.5 mm).

Taking the magnetic saturation and end flux leakage into account, so the accuracy of the FEM is higher than that of analytical method, which can be shown in Table 2.

## 4. Conclusions

The purpose of this paper is to analyze and compare various types of dual layer magnetization patterns systematically and thus provide a general framework for the design of tubular linear machines with similar PM structures. The schematic construction of dual layer magnetization patterns are presented. The analytical models for different types of dual layer magnet arrays are derived from magnetic Laplace’s and Poisson’s equations and boundary conditions. Numerical computation is whereafter conducted to compare the flux field of six typical dual layer magnet arrays. It shows that the dual layer Halbach array can generate high and constant radial flux component, whereas axial-Halbach magnetization can produce high peak value of flux density distribution. A research prototype of tubular linear machine based on dual layer Halbach array and an experimental testbed have been developed, and experimental investigation on magnetic field distribution is conducted. Both numerical simulation and experimental measurement validates the derived analytical model of magnetic field well. The study in this paper can be used for the design optimization and control implementation of tubular electromagnetic linear motors in the future.

## Figures and Tables

**Table 1:** Design parameters of research prototype.

Motor length *L*	250 mm
Maximum radius $R_{o}$	50 mm
Width of radial PM $\tau_{r}$	5 mm
Pole-pitch $\tau_{p}$	25 mm
Number of poles *n*	9
Air gap length *g*	1 mm
Outer rad of external PM $R_{s}$	45 mm
Inner rad of external PM $R_{b}$	32 mm
Outer rad of internal PM $R_{a}$	23 mm
Inner rad of internal PM $R_{r}$	13 mm
Number of winding turns	60

**Table 2:** The comparison of accuracy.

No.	FEM (T)	Analytical Method (T)	Experimental Method (T)
1	−0.7324	−0.7982	−0.7183
2	−0.7882	−0.7830	−0.7725
3	−0.7910	−0.8014	−0.7882
4	−0.6902	−0.7025	−0.6216
5	−0.1413	−0.1408	−0.1425
6	0.3922	0.3919	0.3936
7	0.8381	0.8205	0.8194
8	0.7914	0.7944	0.7923

## Abbreviations

The following abbreviations are used in this manuscript:

FEM	Finite element methom
PM	Permanent magnet
PC	Personal computer
